# Supplementary material for: Antiviral Effect of hBD-3 and LL-37 during Human Primary Keratinocyte Infection with West Nile Virus
Source: Viruses. 2022 Jul 15;14(7):1552. doi: 10.3390/v14071552 (PMC9319560; doi:10.3390/v14071552)
Supplement: Supplementary file 1 [file viruses-14-01552-s001.zip › viruses-1754195-supplementary.pdf]

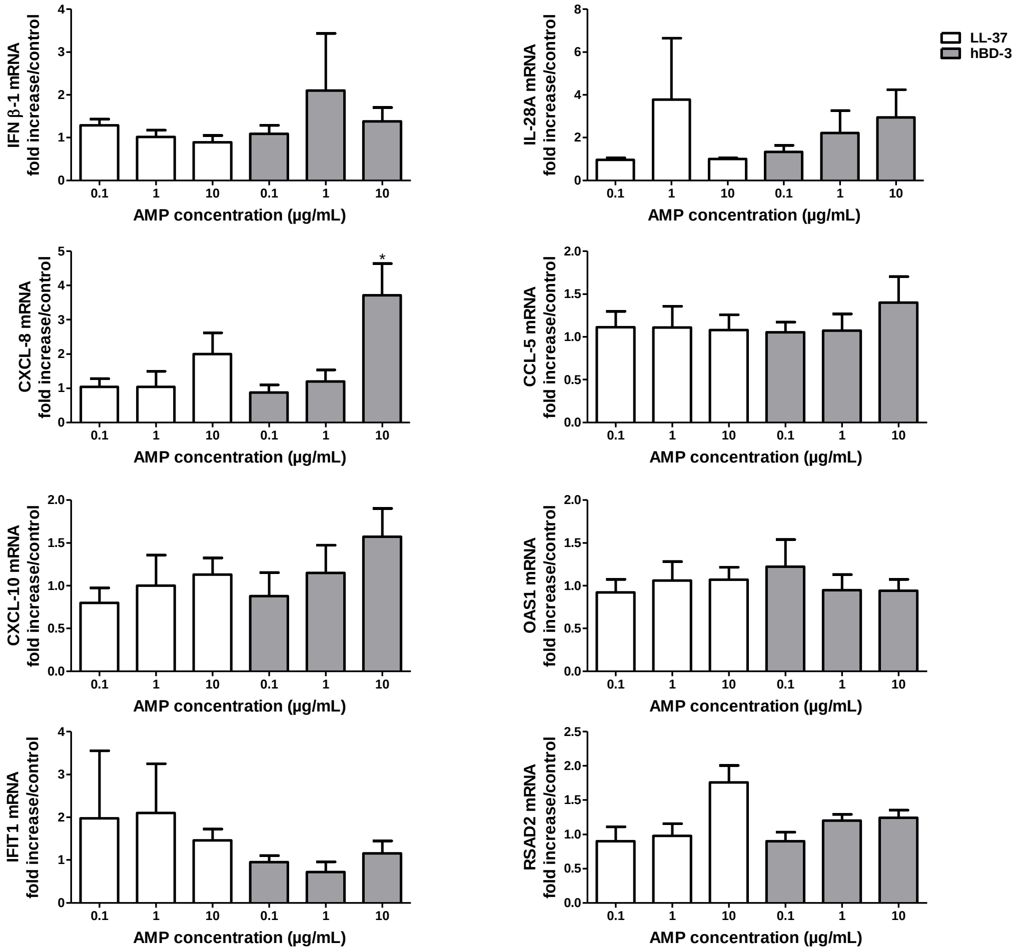


**Figure S1.** Inflammatory response of NHEK stimulated with increasing concentrations of LL-37 or hBD-3 for 3 h. Mean +/- SEM of 6 independent experiments are presented here. $^{*}p < 0.05$.
